# Cardiac Biomarkers and Exercise Training in People With Diabetes: When a Negative Is a Positive∗

**DOI:** 10.1016/j.jacadv.2022.100193

**Published:** 2023-01-27

**Authors:** Michael E. Hall, Affan M. Rizwan, Arsalan Hamid

**Affiliations:** Department of Medicine, University of Mississippi Medical Center, Jackson, Mississippi, USA

**Keywords:** diabetes, exercise, troponin

**Figure:**
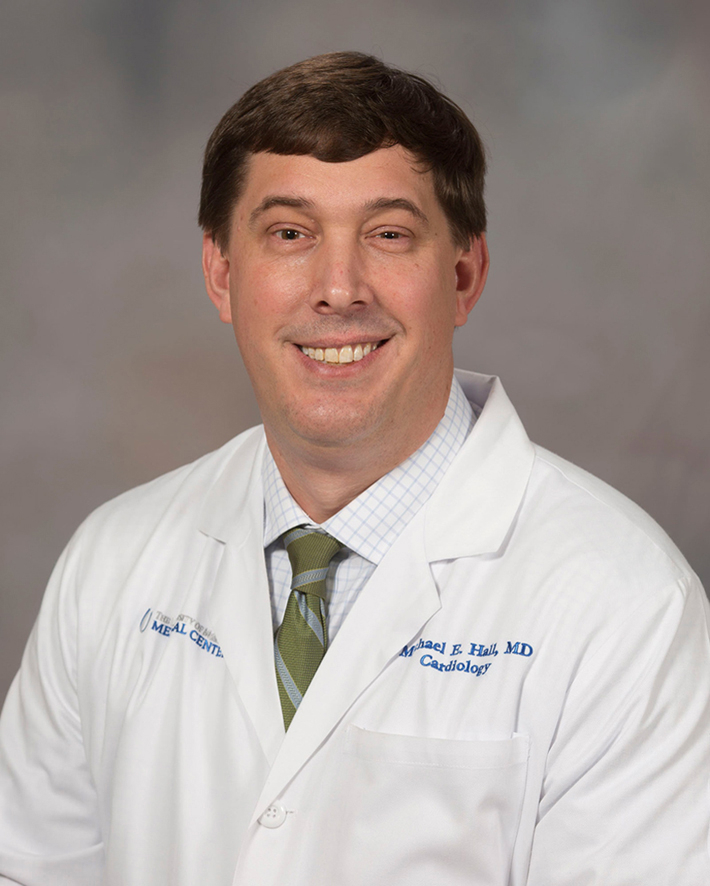

N-terminal proB-type natriuretic peptide (NT-proBNP) and high-sensitivity cardiac troponin T (hs-cTnT) are biomarkers associated with subclinical cardiovascular (CV) remodeling, risk of incident heart failure (HF), and mortality. Patients with type 2 diabetes (T2D) are at increased risk of subclinical CV damage and HF. Physical inactivity is a well-established modifiable risk factor for CV disease (CVD), and increased physical activity is associated with reduced mortality risk. However, conflicting data exist on the association of exercise training with NT-proBNP and hs-cTnT concentrations and their relationship with cardiorespiratory fitness (CRF), particularly in adults with T2D.

In this issue of *JACC: Advances*, Patel et al^1^ report the effects of exercise training on cardiac biomarkers in adults with T2D. This secondary analysis of the HART-D (Health Benefits of Aerobic and Resistance Training in Individuals with Type 2 Diabetes) trial included 135 participants randomized into 3 supervised exercise groups: 1) aerobic training only; 2) resistance training only; or 3) a combination of aerobic and resistance training. These groups were compared to a nonexercise control group (n = 31). The exercise groups were well controlled, achieving around 140 to 145 minutes of exercise time/week (570-680 METS-min/week) based on current physical activity guideline recommendations.^2^ The nonexercise control group maintained baseline physical activity levels throughout the study period (4,180-4,376 steps/week). Cardiac biomarkers (hs-cTnT and NT-proBNP) and CRF measured by peak oxygen uptake (VO_2peak_) were assessed at baseline and after 9 months of exercise.

Exercise training (all 3 exercise groups were pooled) did not significantly change hs-cTnT or NT-proBNP levels compared to the control group over the 9-month study period. Further analyses of the individual exercise groups demonstrated similar findings. They also evaluated the associations of hs-cTnT and NT-proBNP levels with VO_2peak_ and observed that in the exercise training group, higher baseline hs-cTnT was inversely associated with a change in VO_2peak_ but NT-proBNP was not.

This secondary analysis of the HART-D study demonstrates several important points. While the primary intent of the study was to determine if exercise training can reduce cardiac biomarker levels that have been associated with subclinical CVD and incident HF, they also demonstrated that exercise training is not associated with detrimental *increases* in cardiac biomarkers. Increases in troponin levels have been documented from participants in endurance exercise events as well as in those performing shorter-duration high-intensity exercise.^3^ In a study of older adults, walking 30 to 55 km increased troponin I levels in 9% of participants to concentrations >0.040 μg/L (99th percentile), and 27% of those participants died or had a major adverse CV event during a 43-month follow-up.^4^ These findings suggest there may be some underlying myocardial vulnerability in these individuals and that elevated postexercise troponin concentrations may predict future CVD events. The current study observed no increases in serial troponin concentrations after 9 months of moderate-intensity exercise training. This is particularly important given the beneficial impact of lifestyle interventions including increased physical activity for preventing HF in people with T2D.^5^ In observational studies, higher troponin concentrations are associated with incident HF, and higher physical activity levels are associated with a lower risk of incident HF with a preserved ejection fraction (HFpEF). Even in higher risk participants with elevated troponin concentrations, higher levels of physical activity mitigate the risk of HFpEF.^6^ Taken together, clinicians should feel comfortable recommending exercise regimens to improve CVD outcomes in people at increased risk such as those with T2D.

In the present study, the authors did not observe differences in cardiac biomarker levels in any of the exercise groups. However, they did note a greater proportion of participants in the combination aerobic and resistance training group among those with improved CRF (increased VO_2peak_ over 9 months). In overweight and obese adults with T2D in the Look AHEAD trial, intensive lifestyle interventions led to improvements in glycemic control, blood pressure, and reductions in central adiposity, all factors associated with increased risk of HFpEF. Recently Brubaker et al^7^ demonstrated improvements in CV structure and function with caloric restriction and aerobic exercise training in older obese individuals with HFpEF. The addition of resistance training did not significantly increase the exercise capacity (VO_2peak_) or quality of life. However, in the initial analysis of the HART-D study, the combination of aerobic and resistance exercise training was the only intervention which significantly reduced hemoglobin A1c, improved CRF, and reduced body mass (mean reduction of 1.5 kg).^2^ Typically, the physical activity levels in the current study (<150 min/week) produce little to no weight loss, whereas targeting >150 min/week of physical activity is associated with significant weight loss.^8^ Thus, further investigation of the impact of different exercise training regimens on HF risk is warranted, especially in those with T2D, hypertension, or increased visceral adiposity. While increased physical activity and improved CRF are inversely associated with HF risk, whether exercise regimens should be titrated to achieve significant weight loss to reduce HF risk has not been adequately addressed.

In the ARIC (Atherosclerosis Risk In Communities) study, poor physical activity and obesity were associated with elevated hs-cTnT, and this association was attenuated in participants with obesity and higher levels of physical activity.^9^ This relationship is consistent with the findings in the current study, which may explain why there was only an association of CRF with hs-cTnT but not with NT-proBNP. Furthermore, NT-proBNP levels are often inappropriately lower in people with obesity. Therefore, these biomarkers can present unique challenges when tracking subclinical CVD and HF risk particularly in people with obesity. This may be mitigated by evaluating serial biomarker trends over several measurements for a more robust assessment.

Participants in the HART-D study underwent moderate continuous exercise training. High-intensity interval training is associated with improvements in cardiometabolic outcomes.^10^ However, a recent trial demonstrated no significant difference in VO_2peak_ after 3 or 12 months of high-intensity or moderate continuous training compared with guideline-based physical activity.^11^ Further investigation of the long-term impact of exercise training types and intensities in patients with increased risk of HF is warranted.

There were a few limitations in this study including limited follow-up duration and relatively low hs-CTnT concentrations. Nine months of exercise training may not be long enough to modify CV changes that occur over many years in people with T2D. Overall, hs-CTnT and NT-proBNP levels were very low at baseline (mean of 3 ng/L and 18 pg/L, respectively, in the pooled exercise group), and baseline hs-CTnT concentrations were below the lower limit of detection (6 ng/L) in more than half of included participants. Thus, achieving significant reductions in biomarker levels may not be possible when many are undetectable at baseline.

Strengths of this article include comparisons of several exercise training interventions that were highly standardized in a population with T2D and obesity which increase the risk of HF. The findings of this study are reassuring that exercise interventions are safe in people with T2D and are not associated with biomarker elevations linked with subclinical and clinical CVD. Furthermore, patients with T2D being considered for exercise interventions may benefit from an hs-cTnT assessment prior to the initiation of an exercise regimen to “benefit stratify” them. Future research opportunities include studies of higher-risk people with higher baseline concentrations of biomarkers, followed by several visits in the long term to better assess whether exercise training can reduce biomarker levels associated with CVD.

## Funding support and author disclosures

The authors have reported that they have no relationships relevant to the contents of this paper to disclose.
